# A Distributed Chemosensory Circuit for Oxygen Preference in C. elegans

**DOI:** 10.1371/journal.pbio.0040274

**Published:** 2006-08-15

**Authors:** Andy J Chang, Nikolas Chronis, David S Karow, Michael A Marletta, Cornelia I Bargmann

**Affiliations:** 1 Howard Hughes Medical Institute and Laboratory of Neural Circuits and Behavior, The Rockefeller University, New York, New York, United States of America; 2 Graduate Program in Cellular and Molecular Biology, University of Michigan, Ann Arbor, Michigan, United States of America; 3 Departments of Chemistry and Molecular and Cell Biology, University of California Berkeley, Berkeley, California, United States of America; 4 Division of Physical Biosciences, Lawrence Berkeley National Lab, Berkeley, California, United States of America; University of Cambridge, United Kingdom

## Abstract

The nematode Caenorhabditis elegans has complex, naturally variable behavioral responses to environmental oxygen, food, and other animals. C. elegans detects oxygen through soluble guanylate cyclase homologs (sGCs) and responds to it differently depending on the activity of the neuropeptide receptor NPR-1: *npr-1(lf)* and naturally isolated *npr-1(215F)* animals avoid high oxygen and aggregate in the presence of food; *npr-1(215V)* animals do not. We show here that hyperoxia avoidance integrates food with *npr-1* activity through neuromodulation of a distributed oxygen-sensing network. Hyperoxia avoidance is stimulated by sGC-expressing oxygen-sensing neurons, nociceptive neurons, and ADF sensory neurons. In *npr-1(215V)* animals, the switch from weak aerotaxis on food to strong aerotaxis in its absence requires close regulation of the neurotransmitter serotonin in the ADF neurons; high levels of ADF serotonin promote hyperoxia avoidance. In *npr-1(lf)* animals, food regulation is masked by increased activity of the oxygen-sensing neurons. Hyperoxia avoidance is also regulated by the neuronal TGF-β homolog DAF-7, a secreted mediator of crowding and stress responses. DAF-7 inhibits serotonin synthesis in ADF, suggesting that ADF serotonin is a convergence point for regulation of hyperoxia avoidance. Coalitions of neurons that promote and repress hyperoxia avoidance generate a subtle and flexible response to environmental oxygen.

## Abstract 2

A systematic comparison of the cells and genes in *C. elegans* that mediate avoidance of high oxygen levels reveals a distributed network of neurons that promote aerotaxis and those that suppress it in the presence of food.

## Introduction

Sensory systems are shaped by evolution to detect the salient features of an animal's natural environment. At the simplest level, a sensory system is tuned to the most informative cues: specific ultrasonic wavelengths in the bat's echolocation system, or statistically likely features of natural scenes in the vertebrate visual system [1,2]. At more complex levels of processing, the nervous system sets behavioral priorities based on context. Even simple animals generate distinct behaviors to the same sensory cue depending on the other cues that are present at the same time. Food, hunger, danger, and sleep elicit modulatory contexts that change the behavioral output to the same sensory input [3]. Understanding behavior requires an understanding of the strategies that integrate context into sensory responses during ecologically relevant behaviors.

Oxygen is unevenly distributed in soil and water environments [4–6], and is an important sensory cue for many organisms. Animals monitor both internal and environmental oxygen levels to maintain oxygen homeostasis [7]. Either hypoxia or hyperoxia is disruptive: molecular oxygen is essential for many biological processes, but excess oxygen promotes the production of toxic reactive oxygen species [8]. The best-understood mechanism of oxygen homeostasis is the HIF-1 (hypoxia-inducible transcription factor) system, which is conserved in all metazoa [9]. This relatively slow transcriptional pathway for oxygen homeostasis is supplemented by rapid neuronal mechanisms for oxygen sensation. In mammals, the carotid body senses reduced blood oxygenation to mediate a hyperventilatory response and strong arousal [10]. In the nematode *Caenorhabditis elegans,* certain chemosensory neurons detect environmental oxygen to generate aerotaxis behaviors in oxygen gradients [11]. These neurons, called URX, AQR, and PQR, express five genes that encode soluble guanylate cyclase homologs (sGCs) with similarity to the NO-regulated sGCs from other animals. The sGC homolog GCY-35 is required for normal aerotaxis and contains a heme domain that binds molecular oxygen [11], suggesting that oxygen regulates GCY-35 and cyclic guanosine monophosphate (cGMP) production during aerotaxis. A second sGC homolog encoded by the *gcy-36* gene is also needed for aerotaxis [12]. cGMP is thought to open a cGMP-gated sensory transduction channel encoded by the *tax-4* and *tax-2* genes [11]. Through the activity of the sGC homologs and the cGMP-gated channel, animals accumulate at a preferred oxygen concentration of about 10% oxygen.

The behavioral response of C. elegans to oxygen is regulated by natural polymorphisms in a neuropeptide receptor gene, *npr-1* [11,12]. *npr-1* encodes a G protein–coupled receptor related to mammalian neuropeptide Y receptors, and exists in two allelic forms that differ at a single animal acid, NPR-1(215V) and NPR-1(215F) [13]. Both alleles are widely distributed in wild-caught C. elegans populations. Genetic analysis and biochemical characterization of ligand responses indicate that the 215V allele has high activity and the 215F allele has low activity [14,15]. *npr-1(215F)* and *npr-1(lf)* animals have strong aerotaxis responses in oxygen gradients both in the presence of food and in its absence. *npr-1(215V)* animals, including the standard lab strain N2, have strong aerotaxis responses in the absence of food, but fail to avoid high oxygen when food is present [11]. *npr-1(lf)* and *npr-1(215V)* also differ in their locomotory responses to oxygen; reducing oxygen results in slowing of *npr-1(lf),* but not *npr-1(215V),* animals [12]. Slowing requires *gcy-35* and *gcy-36,* and is suppressed by expression of *npr-1(215V)* in URX, AQR, and PQR [12]. These results suggest that *npr-1(215V)* activity suppresses sGC-dependent oxygen responses in the presence of food.

The URX, AQR, and PQR oxygen-sensing neurons have an important role in a second behavior, the naturally polymorphic social feeding behavior of *C. elegans.* Animals with *npr-1(215F)* or *npr-1(lf)* alleles aggregate into feeding groups on bacterial lawns, a behavior that requires *gcy-35* and *gcy-36* in URX, AQR, and PQR neurons [13,16]. Because aggregation is stimulated at high oxygen levels and accumulation into aggregates decreases local oxygen levels, it is likely that hyperoxia avoidance by URX, AQR, and PQR is a key component of aggregation [11,17].

An unanswered question is the mechanism by which *npr-1* activity is integrated into the circuit that responds to food and oxygen. *npr-1(215V)* action in URX, AQR, and PQR neurons suppresses aggregation on food [14]. The simplest interpretation of this result would be that *npr-1(215V)* is an internal “food sensor”—a molecule that inhibits URX, AQR, and PQR activity when food is present. However, many other genes and cells can affect aggregation behavior. For example, aggregation in *npr-1(lf)* animals requires the function of transient receptor potential channel, vanilloid (TRPV) genes *osm-9* and *ocr-2* and the receptor chaperone gene *odr-4* in ASH and ADL nociceptive neurons [18]. Aggregation is also inhibited by the TGF-β gene *daf-7,* which is expressed mainly in a different class of sensory neurons called ASI [18–21]. Given the close relationship between genes and neurons involved in aggregation and hyperoxia avoidance, these other pathways are also candidate regulators of oxygen responses. Indeed, a recent study of oxygen responses in the *npr-1(lf)* background showed that *ocr-2* and the ASH nociceptive neurons regulate aerotaxis and oxygen-dependent locomotory responses [17].

Other than *gcy-35* and *tax-4,* the genes and neurons required for aerotaxis in the food-regulated *npr-1(215V)* background are unknown. Here we undertake a systematic comparison of the cells and genes that mediate hyperoxia avoidance in *npr-1(215V)* and *npr-1(lf)* animals. Our results reveal a distributed network of aerotaxis-promoting neurons and antagonistic neurons that suppress aerotaxis on food. Neuromodulation by food is accomplished by the NPR-1 neuropeptide pathway, noncanonical peptide regulation by the TGF-β–related protein DAF-7, and the neurotransmitter serotonin. Different coalitions of neurons dominate aerotaxis behavior in different conditions. Thus, complex natural behaviors in *C. elegans,* like behaviors in other animals, are assembled by flexible, distributed neuronal networks.

## Results

### Two Distinct Groups of sGC-Expressing Neurons Promote Hyperoxia Avoidance

**Figure 1:**
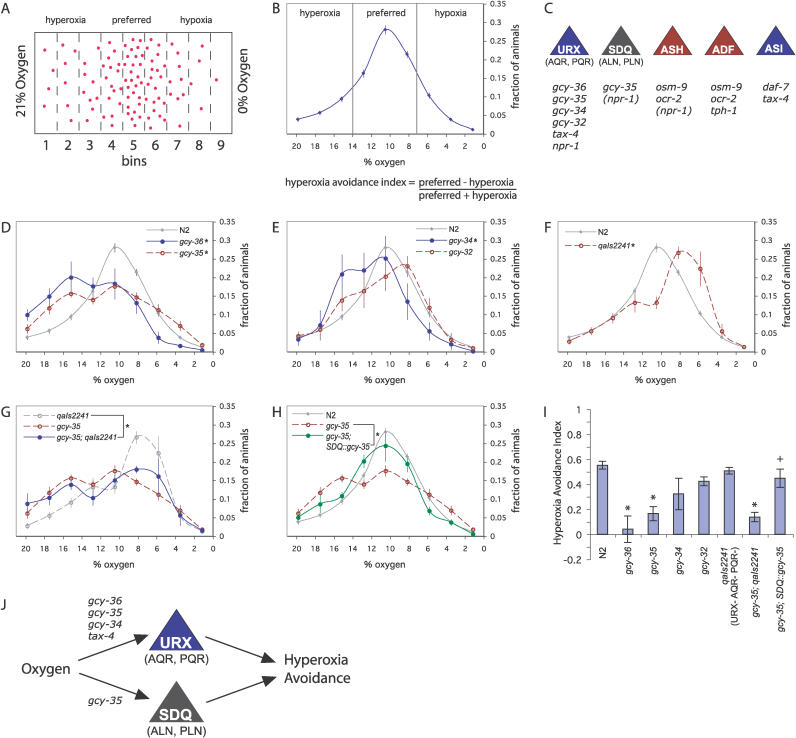
Two Distinct Groups of sGC-Expressing Neurons Promote Hyperoxia Avoidance (A) A cartoon of the typical distribution of 100 wild-type N2 animals (red dots) in a 0%–21% oxygen gradient. Adult animals are placed on an agar surface under a 3-cm × 1.5-cm PDMS chamber; laminar flow of gases at either end of the chamber generates an oxygen gradient within the chamber. The positions of animals are scored after 25 min. For counting, animals are binned in nine equally spaced regions along the device. (B) In a 0%–21% oxygen gradient, wild-type N2 animals avoid both hyperoxia (14%–21% O_2_) and hypoxia (0%–7% O_2_), preferring the center of the gradient (7%–14% O_2_) (*n =* 28 assays, 80–100 animals/assay). (C) Neurons (top row) and genes that appear in this study. *npr-1* is expressed in SDQ and ASH, but not known to affect their function [13]. (D–E) Aerotaxis of *gcy-35* and *gcy-36* mutants (D) and *gcy-32* and *gcy-34* mutants (E). (F) Aerotaxis of *qaIs2241* animals, which bear a transgene that kills the URX, AQR, and PQR neurons. (G) Aerotaxis of *gcy-35; qaIs2241* mutants. (H) Aerotaxis of *gcy-35* mutants in which SDQ, ALN, and PLN were rescued with a *lad-2::gcy-35* transgene. In (D–H), asterisks denote distributions different by chi-square analysis at *p* < 0.01 from the first distribution in the panel, unless otherwise noted. *n* ≥ 3 assays per genotype, 80–100 animals/assay. Error bars are standard error of the mean (SEM). Aerotaxis assays in all figures follow a standard color code: red and blue colors are used for mutants, green is used for transgenic rescue strains, and gray is used for results repeated from an earlier figure. (I) The hyperoxia avoidance index is defined as [(fraction of animals in 7%–14% O_2_) − (fraction of animals in 14%–21% O_2_)] / (fraction of animals in 7%–21% O_2_). In (I), asterisks denote values different from N2 controls at *p* < 0.01 by Dunnett test. Cross, value different from the *gcy-35* control at *p <* 0.05 by Bonferroni *t* test with N2 and *gcy-35* controls. Error bars denote SEM. (J) Hyperoxia avoidance is promoted by two sets of sGC-expressing neurons: (1) some or all of URX, PQR, and PQR; and (2) some or all of SDQ, ALN, and PLN.

**Figure 2:**
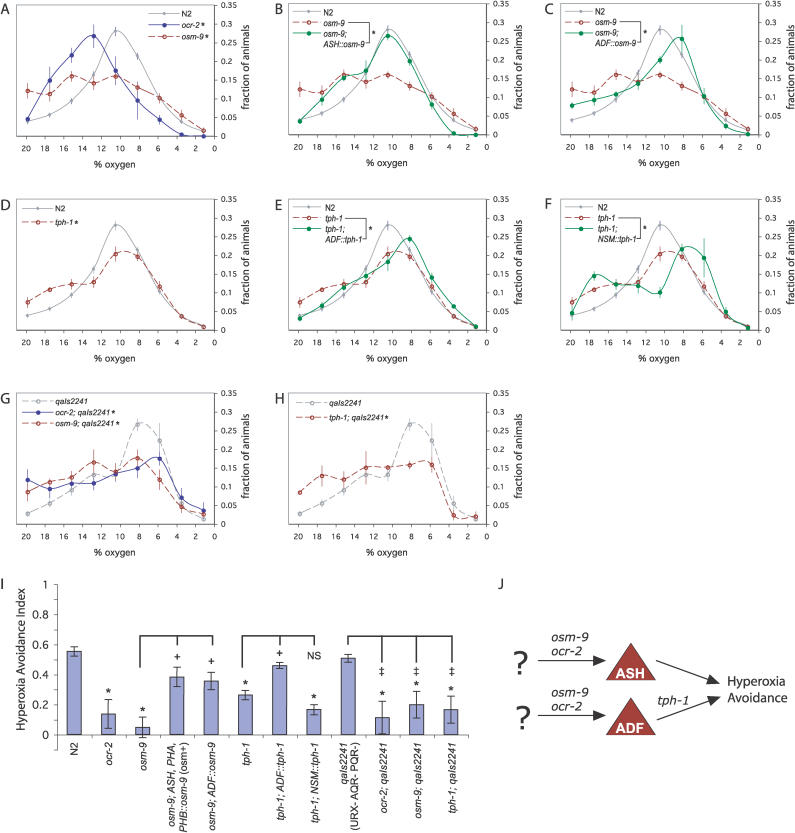
Multiple TRPV-Expressing Neurons Contribute to Hyperoxia Avoidance (A) Aerotaxis of *TRPV* single mutants. The median preferred oxygen concentration of *ocr-2* mutants was significantly higher than N2 (*p <* 0.05 by Dunnett test). (B) Aerotaxis of *osm-9* mutants in which ASH, PHA, and PHB were rescued with an *osm-10::osm-9* transgene [26]. Animals rescued for ASH function were preselected for avoidance of high osmolarity, an ASH behavior (see Materials and Methods). (C) Aerotaxis of *osm-9* mutants in which ADF was rescued by expression from a *cat-1::osm-9* transgene [26]. (D) Aerotaxis of *tph-1* mutants. (E) Aerotaxis of *tph-1* mutants rescued in ADF neurons using a *srh-142::tph-1::gfp* transgene [28]. (F) Aerotaxis of *tph-1* mutants rescued in NSM neurons using a *ceh-2::tph-1::gfp* transgene [28]. (G) Aerotaxis of *TRPV; qaIs2241* double mutants. (H) Aerotaxis of *tph-1; qaIs2241* double mutants. For (A–H), asterisks denote distributions different by chi-square analysis at *p* < 0.01 from the first distribution in the panel, unless otherwise noted. *n* ≥ 3 assays per genotype, 80–100 animals/assay. Error bars denote SEM. (I) Hyperoxia avoidance index as defined in Figure 1. Asterisks, values different from N2 controls at *p* < 0.01 by Dunnett test. Single crosses, values different from the *osm-9* or *tph-1* control at *p <* 0.05 by Bonferroni *t* test with N2 and mutant controls. Double crosses, values different from *qaIs2241* controls at *p <* 0.01 by Dunnett test. NS, not significant. Error bars denote SEM. (J) ASH and ADF sensory neurons promote hyperoxia avoidance through the activity of TRPV channels *osm-9* and *ocr-2.* Serotonin production in ADF by *tph-1,* which is regulated by TRPV channels, also drives this behavior.

**Figure 3:**
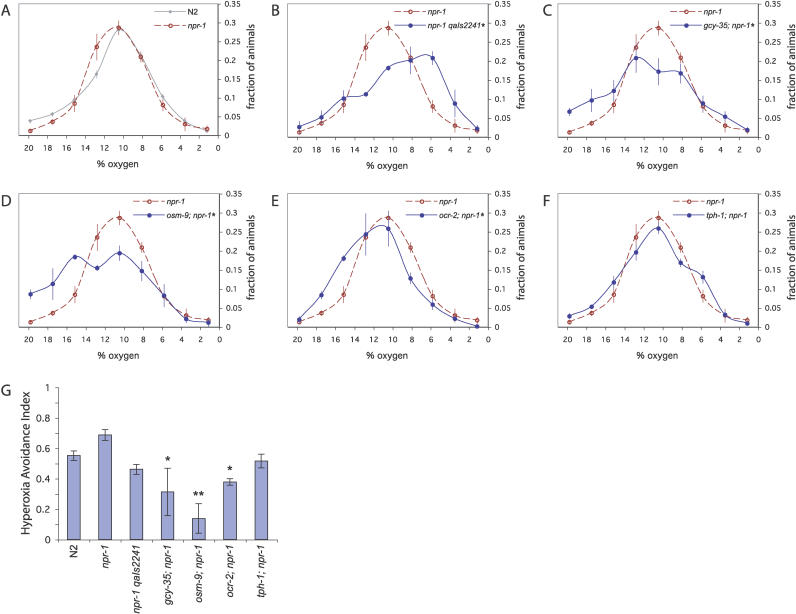
Hyperoxia Avoidance by *npr-1* Mutants Requires sGC and TRPV Activity, but not Serotonin (A) Aerotaxis of *npr-1* mutants. (B) Aerotaxis of *npr-1; qaIs2241* strain. (C) Aerotaxis of *gcy-35; npr-1* double mutants. (D) Aerotaxis of *osm-9; npr-1* double mutants. (E) Aerotaxis of *ocr-2; npr-1* double mutants. (F) Aerotaxis of *tph-1; npr-1* double mutants. For (A–F), asterisks denote distributions different by chi-square analysis at *p* < 0.01 from the first distribution in the panel. *n* ≥ 3 assays per genotype, 80–100 animals/assay. Error bars denote SEM. For (B–E), all double mutants are significantly different from *npr-1* controls, but not significantly different from animals carrying the other mutation (*p* < 0.01 by chi-square analysis of the complete distribution). (G) Hyperoxia avoidance index as defined in Figure 1. Asterisks and double asterisks, values different from *npr-1* controls at *p* < 0.05 and *p* < 0.01, respectively, by Dunnett test. Error bars denote SEM.

**Figure 4:**
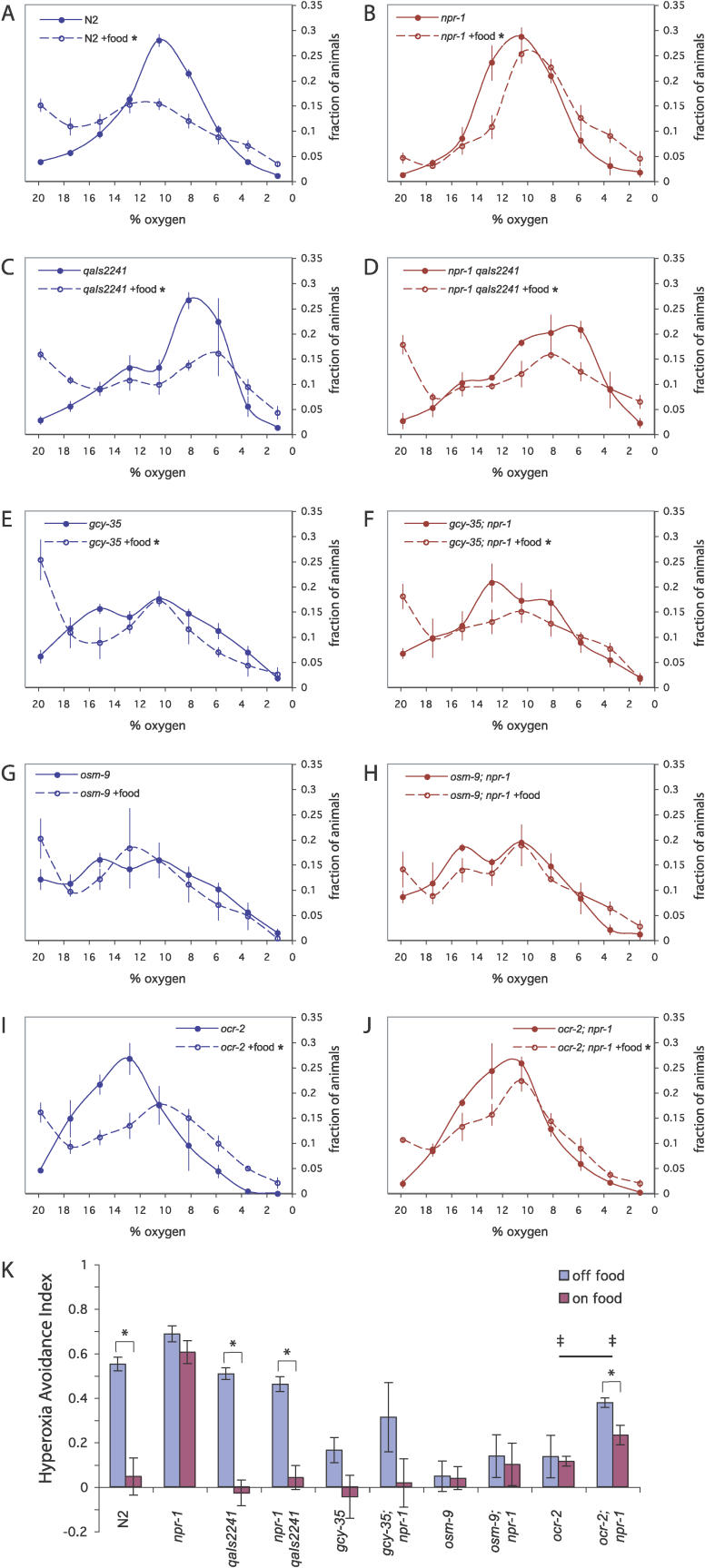
sGC and *ocr-2* TRPV Mutations Restore Food Regulation of Hyperoxia Avoidance to *npr-1* (A–J) In all panels, dotted lines indicate aerotaxis in the presence of a small amount of bacterial food. (A) Aerotaxis of wild-type N2 animals. (B) Aerotaxis of *npr-1* mutants. (C) Aerotaxis of *qaIs2241* strain (URX, AQR, PQR killed). (D) Aerotaxis of *npr-1 qaIs2241* strain. (E) Aerotaxis of *gcy-35* mutants. (F) Aerotaxis of *gcy-35; npr-1* double mutants. (G) Aerotaxis of *osm-9* mutants. (H) Aerotaxis of *osm-9; npr-1* double mutants. (I) Aerotaxis of *ocr-2* mutants. (J) Aerotaxis of *ocr-2; npr-1* double mutants. For (A–J), asterisks denote distributions different by chi-square analysis at *p* < 0.01 from the same genotype without food. *n* ≥ 3 assays per genotype and condition, 80–100 animals/assay. Error bars denote SEM. (K) Hyperoxia avoidance index as defined in Figure 1. Asterisks denote values significantly different from the same genotype without food at *p <* 0.05 by *t* test. Error bars denote SEM. Double crosses indicate that *ocr-2* mutants are significantly regulated by food using chi-square analysis of the entire distribution (*p <* 0.01), and that *ocr-2; npr-1* on food is significantly different from *ocr-2; npr-1* off food and *npr-1* on food (*p <* 0.01 by chi-square analysis) and not significantly different from *ocr-2* mutants on food.

**Figure 5:**
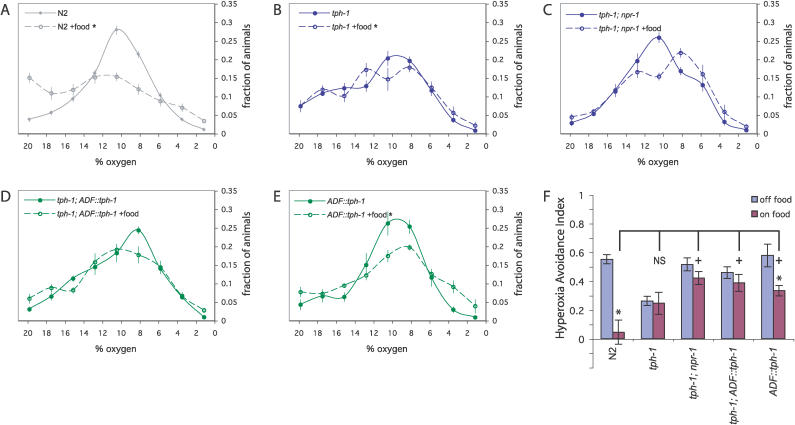
Levels of Serotonin in ADF Neurons Affect Food Regulation of Hyperoxia Avoidance (A–E) In all panels, dotted lines indicate aerotaxis in the presence of a small amount of bacterial food. (A) Aerotaxis of wild-type N2 animals. (B) Aerotaxis of *tph-1* mutants. (C) Aerotaxis of *tph-1; npr-1* double mutants. (D) Aerotaxis of *tph-1* animals rescued in ADF with a *srh-142::tph-1::gfp* transgene [28]. (E) Aerotaxis of wild-type animals expressing *tph-1* in ADF from a *srh-142::tph-1::gfp* transgene. For (A–E), asterisks denote distributions different by chi-square analysis at *p* < 0.01 from the same genotype without food. *n* ≥ 3 assays per genotype and condition, 80–100 animals/assay. Error bars denote SEM. (F) Hyperoxia avoidance index as defined in Figure 1. Asterisks, values significantly different from the same genotype without food at *p* < 0.05 by *t* test. Crosses, values significantly different from N2 on food at *p <* 0.01 by Dunnett test. NS, not significant. Error bars denote SEM.

**Figure 6:**
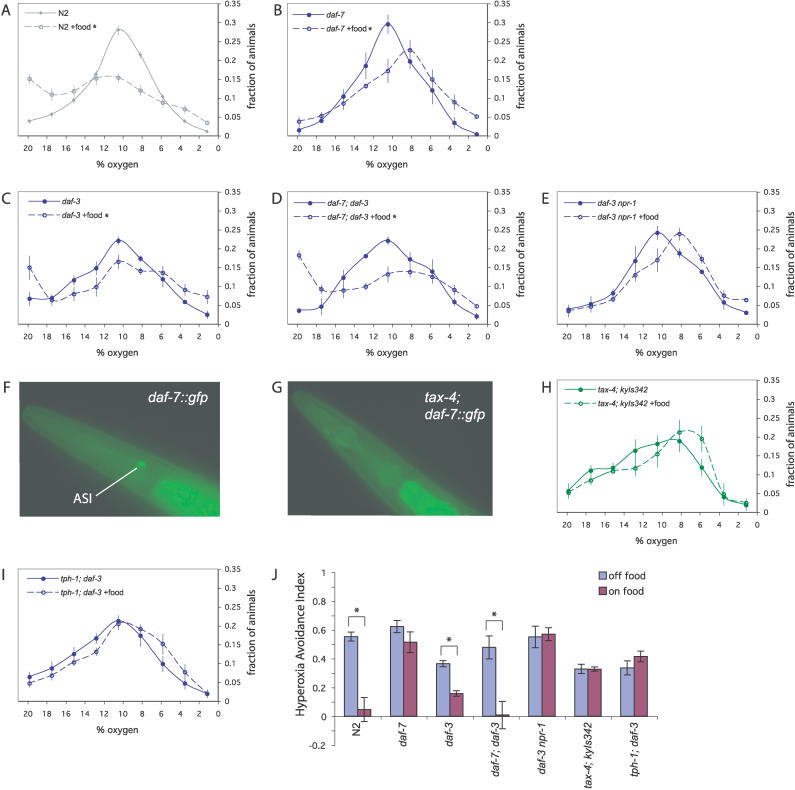
TGF-β Signaling Mediates Food Suppression of Hyperoxia Avoidance (A–E), (H), (I) In all panels, dotted lines indicate aerotaxis in the presence of a small amount of bacterial food. (A) Aerotaxis of wild-type N2 animals. (B) Aerotaxis of *daf-7* mutants. (C) Aerotaxis of *daf-3* mutants. (D) Aerotaxis of *daf-7; daf-3* double mutants. (E) Aerotaxis of *daf-3 npr-1* double mutants. (F–G) *daf-7::GFP* expression in ASI was reduced in a *tax-4* mutant. Anterior is to the left. (H) Aerotaxis of *tax-4; kyIs342* animals, which bear a transgene that rescues *tax-4* in URX, AQR, and PQR, but not in ASI or other neurons. (I) Aerotaxis of *tph-1; daf-3* double mutants. For (A–E), (H), and (I), asterisks denote distributions different by chi-square analysis at *p* < 0.01 from the same genotype without food. *n* ≥ 3 assays per genotype and condition, 80–100 animals/assay. Error bars denote SEM. (J) Hyperoxia avoidance index as defined in Figure 1. Asterisks, values significantly different from the same genotype without food at *p* < 0.05 by *t* test. In the absence of food, no strain is different from N2 controls by Dunnett test. In the presence of food, *daf-7, daf-3 npr-1,* and *tph-1; daf-3* are different from N2 at *p <* 0.01 and *tax-4; kyIs342* different from N2 at *p* < 0.05 by Dunnett test. Error bars denote SEM.

When N2 *npr-1(215V) C. elegans* is placed into a small chamber with a linear gradient from anoxia to atmospheric oxygen in the gas phase, animals rapidly move to an intermediate preferred oxygen concentration between 7%–14% O_2_, avoiding both high and low oxygen levels [11] (Figure 1A and 1B). We found that different genetic and sensory inputs affected the animals' distribution in complex ways (Figures 1–6). Some manipulations caused animals to shift toward higher or lower oxygen but preserved avoidance of oxygen extremes (e.g., Figure 1F); other manipulations resulted in flattened distributions of animals between 7%–21% oxygen but did not affect avoidance of low oxygen (e.g., Figure 1G). Accumulation of animals at one oxygen concentration necessarily affected accumulation at all other concentrations—in other words, individual datapoints within an aerotaxis assay are not independent. Statistical approaches for analyzing aerotaxis data were developed to take the entire distribution into account in representing the mutant responses. Briefly, for each mutant and condition we first compared the complete distribution of animals in the gradient to appropriate control strains using a stringent nonparametric test, chi-square analysis. Distributions that were significantly different at *p* < 0.01 were subjected to further analysis with additional tests, using appropriate corrections for multiple comparisons. The Materials and Methods section includes a full discussion of statistical considerations; primary aerotaxis data are included in Table S1.

In this study, we focused on genes and neurons that contribute to hyperoxia avoidance, the avoidance of oxygen levels above 14% O_2_ (Figure 1B and 1C). Many of the interesting differences between mutants were evident using a simple index for hyperoxia avoidance that compares the fraction of animals in the hyperoxia area (bins 1–3, >14% O_2_) to the fraction in the preferred area (bins 4–6, 7%–14% O_2_). A hyperoxia avoidance index of 1.0 represents complete avoidance of hyperoxia, 0.0 represents no preference, and −1.0 represents a complete preference for hyperoxia. Because each mutant was assayed at least three times, the hyperoxia avoidance index could be used for standard statistical methods such as analysis of variance (ANOVA). In a few cases, the behavior of animals in the gradients showed a significant shift in the preferred oxygen concentration that was not well described by the hyperoxia avoidance index, but could be described using the median preferred oxygen concentration. These cases are discussed individually as they arise. The analysis used here may underreport real differences, but should exclude false-positive results. Throughout this paper, the term “aerotaxis” refers to the complete distribution of animals (Figure 1B), and the phrase “hyperoxia avoidance” refers specifically to the hyperoxia avoidance index (Figure 1I).

Hyperoxia avoidance in N2 *npr-1(215V)* depends on the cGMP-gated channel *tax-4* and the sGC homolog *gcy-35* [11]. Four additional sGC homologs, *gcy-32, gcy-34, gcy-36,* and *gcy-37,* are coexpressed with *gcy-35* and *tax-4* in the URX, AQR, and PQR sensory neurons. Existing loss-of-function mutations in all of these sGCs except *gcy-37* were tested to ask whether other sGCs are involved in oxygen sensing in N2 *npr-1(215V)* animals. *gcy-35* and *gcy-36* mutants were significantly different from wild-type N2 animals based on the hyperoxia avoidance index, whereas *gcy-32* and *gcy-34* mutants were not affected by this measure (Figure 1D, 1E, and 1I). Analysis of the entire aerotaxis distribution showed a significant shift toward higher median preferred oxygen concentrations in *gcy-34* mutants (*p* < 0.05), and a shift in *gcy-32* animals that is unlikely to be significant. In an independent study, *gcy-35* and *gcy-36* were found to affect aerotaxis in the *npr-1(lf)* background, but *gcy-32* and *gcy-34* had no effect under standard assay conditions [12].

The hyperoxia avoidance defects of *gcy-35* and *tax-4* mutants can be rescued by transgenes expressed in the URX, AQR, and PQR neurons [11]. To ask whether these neurons are also necessary for aerotaxis, we expressed the cell-death activator gene *egl-1* [22] under the control of the *gcy-36* promoter to generate a strain in which the URX, AQR, and PQR neurons were absent *(qaIs2241).* No URX, AQR, and PQR neurons could be detected in animals bearing the integrated *qaIs2241* transgene, as assessed either by expression of *gcy-35::GFP* or by Nomarski microscopy (*n* > 100 and *n* = 10, respectively). Surprisingly, the *qaIs2241* strain exhibited robust hyperoxia avoidance that was unlike the sGC mutants *gcy-34, gcy-35,* and *gcy-36* (Figure 1F and 1I). Their hyperoxia avoidance index was normal, but *qaIs2241* animals were shifted to significantly lower oxygen concentrations in a 0%–21% gradient than wild-type animals (*p* < 0.01). These results imply the existence of oxygen-sensing neurons other than URX, AQR, and PQR. They also show that altering sGC gene function leads to qualitatively different results from killing URX, AQR, and PQR neurons (see Discussion).

*gcy-35::GFP* reporter genes are expressed reliably in URX, AQR, PQR, SDQ, ALN, and BDU neurons, and variably in AVM, PLM, and PLN neurons [11,16]. AVM and PLM are mechanosensory neurons; little is known about the functional properties of SDQ, ALN, BDU, and PLN. *gcy-35; qaIs2241* animals were defective in hyperoxia avoidance, unlike *qaIs2241* (Figure 1G and 1I). This result indicates that *gcy-35* has an additional site of action outside the URX, AQR, and PQR neurons. Expressing *gcy-35* in SDQ, ALN, and PLN was sufficient to restore hyperoxia avoidance to *gcy-35* mutants (Figure 1H and 1I), suggesting that SDQ, ALN, and PLN might also serve as oxygen-sensing neurons. Thus, at least two sets of sGC-expressing neurons promote hyperoxia avoidance: (1) some or all of URX, AQR, and PQR; and (2) some or all of SDQ, ALN, and PLN (Figure 1J). The two SDQ neurons are similar in lineage, morphology, and neural connectivity to the AQR and PQR neurons, so they are attractive candidate oxygen sensors.

### The Nociceptive ASH TRPV Neurons and Serotonergic ADF TRPV Neurons Promote Hyperoxia Avoidance

In further screens for effects of sensory mutants on aerotaxis, we found that two C. elegans TRPV sensory channels, *osm-9* and *ocr-2,* affected hyperoxia avoidance in *npr-1(215V)* strains. *osm-9* mutants were highly defective in hyperoxia avoidance; *ocr-2* mutants had significant defects in hyperoxia avoidance, and a shifted median preference towards higher oxygen (*p* < 0.05) (Figure 2A and 2I).

*osm-9* and *ocr-2* function in chemotaxis toward some odors and in avoidance of high osmolarity, nose touch, and chemical repellents [23,24]. The expression of *osm-9* and *ocr-2* overlaps in the chemosensory neurons AWA, ADF, ADL, ASH, PHA, and PHB; *osm-9* is expressed in numerous additional neurons. Several cell-specific transgenes were tested for the ability to rescue *osm-9* hyperoxia avoidance in the N2 *npr-1(215V)* strain. The ASH nociceptive neurons are required for avoidance of chemical, mechanical, and osmotic repellents [25]. Expressing *osm-9* in the ASH nociceptive neurons, together with the posterior PHA and PHB neurons, restored hyperoxia avoidance to *osm-9* mutants (Figure 2B and 2I) [26]. A close correlation was observed between rescue of osmotic avoidance, an ASH-mediated behavior, and rescue of hyperoxia avoidance (see Materials and Methods). These results match with those previously observed in the *npr-1(lf)* background, where expression of OCR-2 in ASH rescued both aggregation and aerotaxis [17,18].

Unexpectedly, a second transgene that rescued an *osm-9* mutant only in ADF neurons also rescued *osm-9* hyperoxia avoidance [26] (Figure 2C and 2I). ADF is a chemosensory neuron that affects formation of the alternative dauer larva stage, chemotaxis towards water-soluble attractants, and olfactory learning [25,27,28]; it has not previously been implicated in oxygen-related behaviors. Unlike the rescuing ASH transgene, the ADF transgene did not rescue osmotic avoidance (see Materials and Methods), and the ASH transgene did not rescue other roles of *osm-9* in ADF [26]. These results suggest that *osm-9* acts in both ASH and ADF neurons to promote hyperoxia avoidance.

The ADF neurons produce serotonin, a neurotransmitter that modulates egg-laying, feeding, locomotion, learning, and other food- and stress-related responses [28–31]. The rate-limiting enzyme for serotonin biosynthesis is tryptophan hydroxylase, which is encoded by the *tph-1* gene in C. elegans [30]*. osm-9* and *ocr-2* mutants have reduced levels of *tph-1* expression in ADF [26]. To ask whether serotonin might contribute to the activity of ADF neurons, we examined *tph-1* null mutants. These animals avoided hyperoxia about half as well as wild-type animals (Figure 2D and 2I). Their intermediate defect is consistent with the idea that ADF functions as one of several neurons that promote hyperoxia avoidance. A transgene that expressed *tph-1* only in the ADF sensory neurons rescued the hyperoxia avoidance defect of *tph-1* mutants (Figure 2E and 2I). A different transgene that expressed *tph-1* in the NSM neurons in the pharynx did not rescue hyperoxia avoidance (Figure 2F and 2I), although it does rescue some effects of *tph-1* on locomotion [28]. This result suggests that serotonin produced by ADF promotes hyperoxia avoidance.

*tph-1* was also required for hyperoxia avoidance in the *qaIs2241* strain that lacked URX, AQR, and PQR neurons, as were *ocr-2* and *osm-9* TRPV genes (Figure 2G–I). These results provide further evidence that TRPV channel–expressing neurons cooperate with sGC-expressing neurons in the oxygen response. The analysis of the sGC and TRPV mutants suggests that no single class of neuron is required for hyperoxia avoidance in N2 *npr-1(215V)* animals. Either of two sets of sGC-expressing neurons can cooperate with either of two sets of TRPV-expressing neurons to generate hyperoxia avoidance in oxygen gradients (Figure 1J and 2J).

### *npr-1(lf)* Strains Can Respond to Food if sGC or *ocr-2* TRPV Activity Is Reduced

The basic aerotaxis responses of *npr-1(lf)* animals were similar to those of N2 *npr-1(215V)* animals [11] (Figure 3A), and most of the cells and genes required for hyperoxia avoidance appeared to be similar. *npr-1(lf)* animals with the *qaIs2241* transgene that killed URX, AQR, and PQR neurons showed robust hyperoxia avoidance, but the aerotaxis distribution was significantly shifted compared to *npr-1(lf)* alone (Figure 3B and 3G). *npr-1(lf)* hyperoxia avoidance was reduced by mutations in *gcy-35, osm-9,* and *ocr-2* (Figure 3C–3E and 3G); similar results were obtained independently by de Bono and colleagues [12,17]. One significant difference between N2 and *npr-1(lf)* was that a *tph-1* mutation did not significantly diminish hyperoxia avoidance in the *npr-1(lf)* strain (Figure 3F and 3G).

The presence of a small amount of bacterial food in the aerotaxis chamber strongly suppressed hyperoxia avoidance of N2 strains [11] (Figure 4A and 4K). In *npr-1(lf)* strains, the hyperoxia avoidance index was nearly unchanged by food [11] (Figure 4B and 4K), and aerotaxis was shifted slightly but significantly toward lower oxygen levels (*p* < 0.05). N2; *qaIs2241* animals in which URX, AQR, and PQR were killed were regulated by food, like N2 animals (Figure 4C and 4K). Hyperoxia avoidance in *npr-1 qaIs2241* animals was also strongly regulated by food, unlike the response of *npr-1(lf)* animals (Figure 4D and 4K). The reappearance of food regulation was surprising, but consistent with observations that oxygen-dependent slowing on food is restored in *npr-1(lf)* animals with *gcy* mutations or hyperpolarized URX, AQR, and PQR neurons [12] (Figure 4E, 4F, and 4K). The recovery of food regulation also supports the model from aggregation studies that the main site of *npr-1* action is the sGC-expressing URX, AQR, and PQR neurons [18], which are not required for food regulation (Figure 4C and 4K).

*osm-9* and *ocr-2* TRPV mutations affected both food regulation and hyperoxia avoidance in the *npr-1(lf)* background. *osm-9* mutants were defective in hyperoxia avoidance in all *npr-1* backgrounds, with or without food (Figure 4G, 4H, and 4K). The hyperoxia avoidance index of *ocr-2; npr-1(lf)* double mutants was significantly regulated by food, unlike that of *npr-1(lf)* mutants (Figure 4I–4K).

The simplest initial model explaining *npr-1* aerotaxis behavior would have been that *npr-1(215V)* is the essential link between food sensation and hyperoxia avoidance, but this model must be incorrect. *npr-1* contributes to food regulation of aerotaxis, but the analysis of double mutants shows that alternative pathways retain covert food sensitivity in *npr-1(lf)* strains.

### Increases and Decreases in ADF Serotonin Affect Hyperoxia Avoidance on Food

Serotonin regulates numerous food-associated behaviors in *C. elegans,* and therefore was considered as a possible regulator of aerotaxis*.* Indeed, in the N2 *npr-1(215V)* strain, the serotonin biosynthesis gene *tph-1* was required both for robust hyperoxia avoidance (Figure 2) and for food regulation: the residual hyperoxia avoidance of *tph-1* mutants was not regulated by food (Figure 5A, 5B, and 5F). Hyperoxia avoidance in *tph-1; npr-1* double mutants was unregulated by food, as observed for each single mutant (Figure 5C and 5F). These results implicate *tph-1* in food regulation in *npr-1(215V)* strains.

The hyperoxia avoidance defect of *tph-1* mutants off food was rescued in *tph-1; ADF::tph-1* transgenic animals (Figure 2), but these rescued animals were not regulated by food (Figure 5D and 5F). These results are consistent with models in which food regulation requires the endogenous *tph-1* promoter, accurate regulation of ADF serotonin levels, or additional serotonergic neurons*.* The *ADF::tph-1* transgene was introduced into wild-type animals to distinguish among possible mechanisms of food regulation. This strain should have increased *tph-1* expression in ADF, and the *srh-142* ADF promoter should be resistant to normal transcriptional pathways for *tph-1* regulation. Hyperoxia avoidance in wild-type animals bearing the *ADF::tph-1* transgene was poorly regulated by food compared to N2 controls (Figure 5E and 5F). Thus regulation of hyperoxia avoidance by food requires appropriate regulation of ADF serotonin: either loss of ADF *tph-1* or unregulated ADF *tph-1* expression disrupted food regulation of *npr-1(215V)* strains. These results point toward *tph-1* in ADF as a possible site of food regulation in the *npr-1(215V)* strain. In particular, they suggest that high levels of ADF *tph-1* generate strong hyperoxia avoidance on or off food.

### *daf-7/*TGF-β Reduces ADF Serotonin to Block Hyperoxia Avoidance on Food

The C. elegans TGF-β homolog DAF-7 responds to food and crowding to regulate formation of the alternative dauer larva stage; low levels of *daf-7* activity are associated with stressful conditions that favor dauer formation [20,21]. *daf-7* mutants aggregate on food, suggesting a possible interaction with oxygen-regulated behaviors [19], but this process is genetically different from *npr-1* aggregation [16,18]. We found that *daf-7* mutants exhibit robust hyperoxia avoidance in the presence or absence of food (Figure 6A, 6B, and 6J). These results suggest that like *npr-1(215V), daf-7* activity suppresses hyperoxia avoidance on food.

During reproductive growth, secreted DAF-7 antagonizes the activity of the SMAD transcription factor DAF-3 in target cells [19,32]. *daf-3* mutants are dauer defective, *daf-7* mutants are dauer constitutive, and *daf-7; daf-3* mutants resemble *daf-3* mutants. Similar epistasis relationships were observed in hyperoxia avoidance. *daf-3* hyperoxia avoidance was regulated by food (Figure 6C and 6J), and hyperoxia avoidance of *daf-7; daf-3* animals was also regulated by food, resembling *daf-3* mutants rather than *daf-7* mutants (Figure 6D and 6J). These results suggest that *daf-7* regulates hyperoxia avoidance through the transcriptional *daf-3/SMAD* pathway. Hyperoxia avoidance in *daf-3 npr-1* mutants resembled that of *npr-1* single mutants and was not regulated by food (Figure 6E and 6J). These results indicate that *npr-1* does not act through the *daf-3* transcriptional pathway.

*daf-7* is expressed most strongly in the ASI sensory neurons, where its transcription is induced by food and suppressed by crowding [21]. This activity-dependent pathway for *daf-7* expression in ASI requires the cGMP-gated sensory channel *tax-4* [33] (Figure 6F and 6G). To ask whether *daf-7* expression in ASI might affect aerotaxis, we examined a strain in which *tax-4* was mutant in ASI and other sensory neurons, but rescued in URX, AQR, and PQR neurons. Hyperoxia avoidance in these strains was not regulated by food (Figure 6H and 6J), suggesting that food could act in part by regulating *daf-7* expression in ASI.

**Figure 7:**
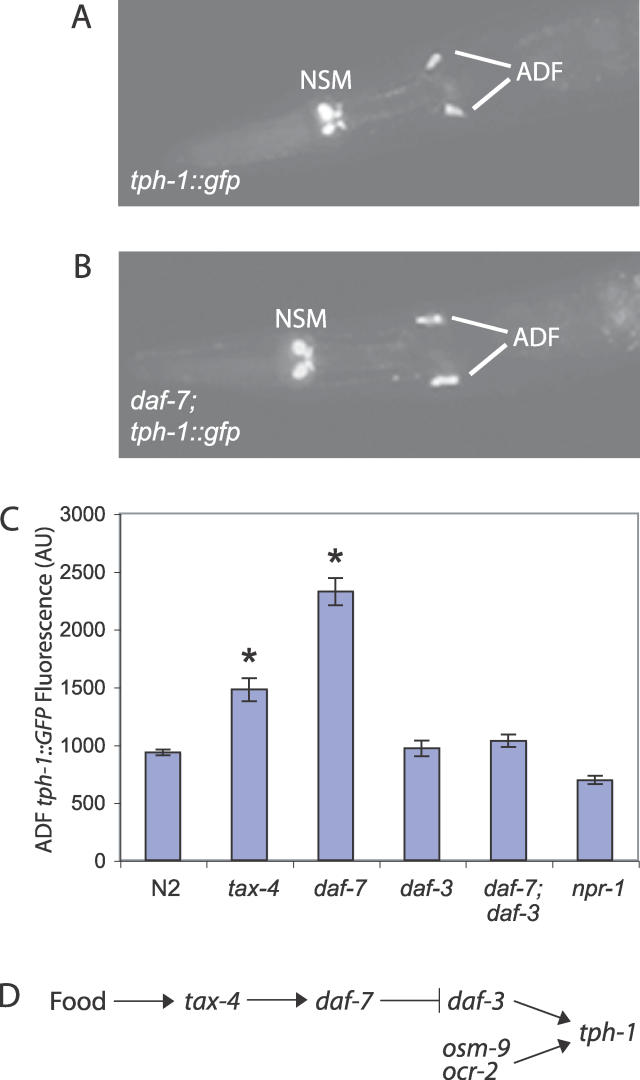
Serotonin Production in ADF Neurons is Regulated by TGF-β Signaling (A–B) *tph-1::GFP* expression in wild-type (A) and *daf-7* (B) adults. Anterior is to the left. Two NSM neurons and two ADF neurons are visible in each animal. (C) Quantitation of *tph-1::GFP* fluorescence in ADF neurons. Asterisks, values different from N2 controls at *p* < 0.01 by Dunnett test. *daf-7* mutants were different from N2, *daf-3*, and *daf-7; daf-3* at *p* < 0.05 by Bonferroni *t* test. *n* ≥ 18 animals per genotype. Error bars denote SEM. (D) Model for food regulation of aerotaxis, combining genetic results from Figure 6 with molecular results from this Figure.

Both *daf-7* and the serotonin biosynthetic enzyme *tph-1* function in food and stress responses, and *tph-1* has complex positive and negative interactions with genes in the dauer formation pathway, including *daf-7* [30,31]*.* We found that *daf-7* mutants had significantly increased *tph-1::GFP* expression in ADF neurons (Figure 7A–7C). *tph-1* is the rate-limiting enzyme for serotonin synthesis, so this induction should increase total ADF serotonin levels and therefore promote aerotaxis. *tph-1::GFP* expression in ADF was also increased in *tax-4* mutants (Figure 7C), which have a low level of *daf-7* expression in ASI. These observations suggest that *tph-1* in ADF might act at a hub between aerotaxis-promoting and aerotaxis-suppressing networks.

Increased ADF serotonin stimulates hyperoxia avoidance on food in N2 *npr-1(215V)* animals (Figure 5E and 5F), so the induction of *tph-1* in *daf-7* mutants could explain why *daf-7* mutants retained strong hyperoxia avoidance on food. This hypothesis was supported by molecular and genetic epistasis results. At a molecular level, *daf-3* suppressed the *tph-1::GFP* induction caused by the *daf-7* mutation (Figure 7C), as well as suppressing *daf-7* hyperoxia avoidance on food (Figure 6D and 6J). These results indicate that *daf-7* activity inhibits *daf-3* to inhibit *tph-1* expression in ADF (Figure 7D). At a genetic level, *tph-1* was epistatic to *daf-3,* so that hyperoxia avoidance in *tph-1; daf-3* double mutants was unregulated by food, as in *tph-1* single mutants (Figure 6I and 6J). These results are consistent with the model that *daf-3* functions by regulating *tph-1* expression. *daf-3* is not required for basal *tph-1* expression in ADF, only for its regulation by *daf-7* (Figure 7C); since *tph-1* is also regulated by TRPV and calcium signaling pathways [26], *daf-3* may not be the only link between food and *tph-1* expression (Figure 7D).

By both molecular and genetic criteria, *daf-7* and *npr-1* appeared to act in separate pathways to regulate hyperoxia avoidance on food. The expression of *tph-1::GFP* in ADF was not increased in *npr-1* animals compared to wild-type animals (Figure 7C), and *daf-3 npr-1* mutants resembled *npr-1* single mutants in hyperoxia avoidance (Figure 6E and 6J), indicating that *daf-3* is not the target for *npr-1.*

**Figure 8:**
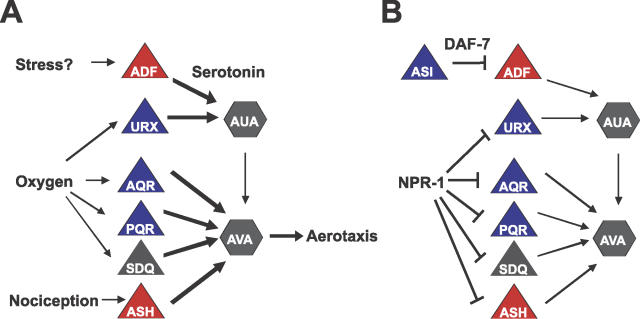
A Distributed Network of Oxygen-Sensing Neurons (A) Aerotaxis-promoting neurons. In the absence of food, parallel networks of sensory neurons generate hyperoxia avoidance. Triangles denote sensory neurons; hexagons denote interneurons. URX, AQR, and PQR*,* and SDQ, ALN, and BDU neurons (abbreviated as “SDQ” for simplicity) express sGCs; these neurons are likely oxygen sensors*.* ASH and ADF neurons express the TRPV channels *osm-9* and *ocr-2;* they might be modulatory neurons, or might respond to oxygen directly. ADF promotes aerotaxis by producing serotonin. Our genetic results suggest that robust aerotaxis requires at least one class of sGC neuron and at least one class of TRPV neuron. Synaptic connections to the AUA interneurons and AVA backward command neurons are shown; additional synapses are omitted [34]. (B) Aerotaxis-suppressing pathways. In the presence of food, the aerotaxis neurons are inhibited by the TGF-β homolog DAF-7 and the neuropeptide receptor NPR-1. Food and *tax-4* activity in ASI neurons stimulate the synthesis of DAF-7, which acts through DAF-3 to inhibit serotonin production in ADF and suppress aerotaxis. Increased or unregulated serotonin expression in ADF allows aerotaxis in the presence of food. Food inhibition may involve additional food-sensing pathways in ADF (perhaps via *osm-9* and *ocr-2*) or serotonin signaling from other food-sensing neurons (such as NSM). NPR-1 is expressed in URX, AQR, PQR, ASH, SDQ, and AUA neurons [14] and could inhibit their function in a food-dependent or food-independent fashion. Many of the signaling pathways and neurons described here have the potential to regulate each other's activity. *tph-1* mutants have decreased *daf-7* expression [30], and *daf-7* affects gene expression in the ASH neurons [47]. A food-related change in serotonin levels affects nociceptive signaling in ASH [38]. The NPR-1 ligand FLP-21 is expressed by ASH and ADL [15]. The regulatory relationships in (B) are supported by the genetics, molecular biology, and behavioral assays in this paper, but these relationships could be reconfigured under different conditions.

These results implicate two different pathways in food regulation of aerotaxis: one mediated by neuropeptide *npr-1* suppression of sGC neurons, and one mediated by *daf-7* suppression of *tph-1* expression in ADF. Neurons that promote and regulate hyperoxia avoidance belong to partly redundant coalitions that antagonize each other: no unique system directs either hyperoxia avoidance or food regulation. A model for this network is presented in Figure 8.

## Discussion

### Oxygen-Sensing Neurons Interact in a Distributed Aerotaxis Circuit

The C. elegans nervous system consists of 302 neurons with reproducible morphologies and synapses [34]. Many of these neurons have characteristic sensory or motor functions [25,35,36]. However, food is able to alter the neurons required for several C. elegans behaviors [37,38], often through serotonin-dependent pathways [28–31]. Our results suggest that the network for oxygen-regulated behavior is flexible and distributed. Like the crustacean feeding circuit, the leech escape circuit, and the mammalian hippocampus, oxygen responses are encoded in the behavior of neuronal populations, not just individual neurons [39–41].

At least four sets of sensory neurons promote avoidance of high oxygen: (1) some or all of URX, AQR, and PQR, which express multiple sGCs; (2) some or all of SDQ, ALN, and PLN, which express the sGC homolog *gcy-35*; and (3–4) the *osm-9* (TRPV)–expressing neurons ADF and ASH (Figure 8A). In the absence of food, either URX, AQR, and PQR neurons or the other sGC neurons can be sufficient for hyperoxia avoidance, although they result in different peak oxygen preferences: about 10% O_2_ for URX, AQR, and PQR, and about 8% O_2_ for the alternative neurons. Avoidance of hypoxia (below 7% O_2_) appears to represent a separate system that is largely independent of these pathways.

At least three predicted soluble guanylate cyclases, *gcy-34, gcy-35,* and *gcy-36,* contribute to hyperoxia avoidance in an N2 background. Mammalian sGCs function as obligate dimers [42], so the possible complement of different cyclases in URX, AQR, and PQR is substantial, perhaps explaining why sGC mutations are different from killing URX, AQR, and PQR. In an independent study, *gcy-35* and *gcy-36,* but not *gcy-32* or *gcy-34,* were found to be required for hyperoxia avoidance in *npr-1* strains [12]. Enhanced aerotaxis activity of URX, AQR, and PQR in *npr-1* mutants may relieve the requirement for *gcy-34* activity. However, even in *npr-1* mutants, either *gcy-32* or *gcy-34* contributes to avoidance of high oxygen levels under sensitized conditions [12].

URX, AQR, PQR, SDQ, ALN, and PLN express sGCs that bind to molecular oxygen, consistent with a primary oxygen-sensing function [11]. We suggest that these neurons are activated by high oxygen to trigger avoidance behavior. An avoidance function is consistent with the neuronal connectivity of URX, AQR, PQR, and SDQ neurons; like ASH and other sensory neurons that mediate avoidance, these neurons synapse onto the command interneurons that regulate the choice between forward and backward movement [34] (Figure 8A).

The TRPV-expressing neurons ADF and ASH also promote hyperoxia avoidance, and an *osm-9* mutant can be rescued by expression in either of those cells. In addition, ASH neurons contribute to aggregation in *npr-1* strains [18]. No direct molecular mediators of oxygen sensation have been defined in ADF and ASH. These neurons might sense oxygen directly through an unknown mechanism, or they might act indirectly to stimulate the activity of the sGC-expressing neurons. ASH neurons are polymodal nociceptors that generate robust calcium transients to noxious stimuli such as high osmotic strength, quinine, and touch [43], but ASH does not respond with strong calcium transients when exposed to decreases or increases in oxygen in the range where hyperoxia avoidance is observed (unpublished data). It is possible that a subtle modulation of ASH activity by oxygen contributes to a distributed oxygen response, or that other noxious signals sensed by ASH converge with oxygen at downstream neurons [18].

The ADF neurons have not been implicated in aggregation, but our results indicate that ADF has a significant role in aerotaxis of *npr-1(215V)* animals. ADF stimulates aerotaxis in the absence of food by producing serotonin. A variety of results suggest that ADF serotonin may be a stress signal in *C. elegans;* this possibility is considered below.

The wiring diagram of synaptic connections in C. elegans suggests that the network of aerotaxis neurons converges on AVA, the command interneuron responsible for generating backward motion; ASH, AQR, PQR, and SDQ all make connections to AVA [34] (Figure 8A). Another likely site of signal integration is the interneuron AUA, which receives most of its synaptic inputs from ADF and URX and synapses onto AVA and other neurons.

### TGF-β Signaling, Serotonin, and Aerotaxis on Food

C. elegans egg-laying, feeding, locomotion, and olfactory behavior are all regulated by food, in part through serotonin-dependent pathways [28–31]. Our results identify serotonin and the TGF-β homolog DAF-7 as interacting modulators of hyperoxia avoidance in *npr-1(215V)* strains. Either decreased or increased serotonin disrupts food regulation: *tph-1* mutants have poor hyperoxia avoidance that is not regulated by food, whereas *daf-7* strains that overexpress *tph-1* have robust aerotaxis that is not regulated by food. Unregulated expression of *tph-1* from the *srh-142* promoter results in food-resistant aerotaxis. These results suggest that *tph-1* expression in ADF functions as a dose-dependent stimulator of hyperoxia avoidance.

Genetic and molecular results indicate that *daf-7* suppresses hyperoxia avoidance on food by repressing *tph-1* expression in ADF neurons. The expression of *daf-7* in ASI is stimulated by food and *tax-4,* providing a link between food availability and *daf-7* activity [21,33]. Thus, the *daf-7/tph-1* regulatory loop may be a component of normal food regulation of hyperoxia avoidance. It is possible that other serotonergic neurons affect food regulation as well.

*tph-1* expression in ADF can be induced by infection with bacterial pathogens, high temperature, calcium signaling, and genes in the dauer formation pathway [26,28,31]. High temperature and pathogenic infection are natural stressors for *C. elegans,* and the dauer pathway is a general physiological response to stress states, suggesting that induction of serotonin in ADF is a common stress-related response. Hyperoxia is a metabolic stressor and a mutagen; the nociceptive ASH neurons and the ADF serotonergic neurons may function to stimulate hyperoxia avoidance (and thus decrease oxidative stress) in the presence of other environmental or metabolic stressors.

Paradoxically, serotonin can substitute for the effects of food in many behavioral assays [29], and therefore may communicate either food or stress signals depending on the amount of serotonin or the neuronal source of serotonin*.* An interesting contrast to hyperoxia avoidance is provided by ASH nociception, which is stimulated by serotonin and heightened in the presence of food, the condition in which hyperoxia avoidance is suppressed [38]. The behaviors associated with serotonin signaling—enhancement of feeding or egg-laying, enhanced slowing on food, alteration of nociceptive pathways, and olfactory learning—are all highly sensitive to the transition between well-fed and starved states [28,29,37,38,44]. ADF serotonin release might be used to signal and enhance the transition between these states, rather than representing either the positive or the negative sensory context.

### Aerotaxis, Social Feeding, and Foraging: Flexibility and Plasticity of Natural Behavior

By repressing the activity of oxygen-sensing neurons [12,17], *npr-1(215V)* activity in N2 strains enhances the food sensitivity of an existing oxygen-sensing network regulated by *daf-7* and *tph-1.* The phenotypes of *npr-1(lf)* strains are consistent with increased URX, AQR, and PQR activity, and subsequent stimulation of both hyperoxia avoidance and aggregation on food. C. elegans consumes oxygen, so oxygen levels within an aggregate of C. elegans are lower than those in the surrounding bacterial lawn ([17]; M. Peliti, personal communication). Aggregation only occurs in strains that maintain strong hyperoxia avoidance in the presence of food (Table S3). These results suggest that aggregation behavior is partly driven by hyperoxia avoidance, and that animals within an aggregate cooperate to lower ambient oxygen to a preferred level. Aggregation in microaerophilic flagellates has been shown to have a similar oxygen-lowering effect [45].

More generally, hyperoxia avoidance may represent a foraging behavior in which C. elegans searches for the lower oxygen concentrations that correspond to food. In the soil, pockets of bacteria growing on decaying organic matter create local sinks of oxygen; even Escherichia coli colonies on an agar surface consume oxygen faster than it can diffuse through the lawn [11]. N2 *npr-1(215V)* animals suppress the drive to find low oxygen in the presence of adequate E. coli food, a strategy that may allow them to exploit a greater range of environments. Thus the recent evolutionary appearance of C. elegans strains with *npr-1(215V)* activity may have added flexibility to C. elegans feeding behavior [15].

The analysis of wild-type and *npr-1* animals on and off food shows that despite its stereotyped neuroanatomy, C. elegans can generate hyperoxia avoidance using flexible configurations of neurons. The preferred oxygen concentration can be altered by changing the class of sensory neurons that dominate the response, or by changing the properties of the sensory neurons. The strength of the avoidance response can be regulated by different food signals that modulate oxygen-sensing circuits. Our results suggest that the simple nervous system of C. elegans has the potential to assemble alternative functional circuits analogous to the dynamic modulated networks in crustacean feeding circuits, leech escape response circuit, or hippocampal place cells [39–41]. The complexity and subtlety of these pathways suggests that oxygen—a highly variable stimulus in the soil environment—is an important regulator of natural C. elegans behavior.

## Materials and Methods

### Strains.

Strains were cultured under standard conditions [46] and fed E. coli HB101. Wild-type animals were C. elegans Bristol strain N2. Other strains used in this work include: AX1297 *gcy-36 (db66)* X, CX6448 *gcy-35 (ok769)* I, RB1062 *gcy-34 (ok1012)* V, CX6804 *gcy-32 (ok995)* V, CX7102 *lin-15 (n765) qaIs2241 [gcy-36::egl-1, gcy-35::gfp, lin-15(+)]* X, CX7104 *gcy-35 (ok769)* I; *qaIs2241* X, CX8142 *gcy-35 (ok769)* I; *kyEx1248 [lad-2::gcy-35, unc-122::dsRed],* CX4544 *ocr-2 (ak47)* IV, CX4537 *osm-9 (ky10)* IV, CX7265 *osm-9 (ky10)* IV; *yzEx53 [osm-10::osm-9, elt-2::gfp],* CX7264 *osm-9 (ky10)* IV; *yzEx51 [cat-1::osm-9, elt-2::gfp],* CX7456 *ocr-2 (ak47)* IV; *qaIs2241* X, CX7250 *osm-9 (ky10)* IV; *qaIs2241* X, DA609 *npr-1 (ad609)* X, CX7158 *npr-1 (ad609) qaIs2241* X, CX7157 *gcy-35 (ok769)* I; *npr-1 (ad609)* X, CX4821 *osm-9 (ky10)* IV; *npr-1 (ad609)* X, CX4649 *ocr-2 (ak47)* IV; *npr-1 (ad609)* X, CB1372 *daf-7 (e1372)* III, CB1376 *daf-3 (e1376)* X, JT5464 *daf-7 (e1372)* III; *daf-3 (e1376)* X, CX7926 *daf-3 (e1376) npr-1 (ad609)* X, DR1808 *mIs6 [daf-7::gfp, rol-6(su1006)]* X, CX7394 *tax-4 (ks28)* III; *mIs6* X, CX6858 *tax-4 (ks28)* III; *kyIs342 [gcy-32::tax-4::gfp, unc-122::gfp],* GR1321 *tph-1 (mg280)* II, CX7749 *tph-1 (mg280)* II; *kyEx1087 [ceh-2::tph-1::gfp, unc-122::gfp],* CX76741 *tph-1 (mg280)* II; *kyEx953 [srh-142::tph-1::gfp, unc-122::gfp],* CX8060 *kyEx953*, CX7888 *tph-1 (mg280)* II; *qaIs2241* X, CX8151 *tph-1 (mg280)* II; *npr-1 (ad609)* X, GR1334 *tph-1 (mg280)* II; *daf-3 (mgDf90)* X, GR1333 *yzIs71 [tph-1::gfp, rol-6(su1006)]* V, CX7248 *tax-4 (ks28)* III; *yzIs71* V, CX8013 *daf-7 (e1372)* III; *yzIs71* V, CX8012 *yzIs71* V; *daf-3 (e1376)* X, CX8059 *daf-7 (e1372)* III; *yzIs71* V; *daf-3 (e1376)* X, and CX8105 *yzIs71* V; *npr-1 (ad609)* X. In all cases, null alleles or strong loss-of-function alleles were used.

### Molecular biology.

A *lad-2::gcy-35*-rescuing plasmid was constructed by PCR amplification of the *lad-2* promoter from genomic DNA and insertion into FseI to AscI in an expression vector containing *gcy-35* cDNA in pSM1, a modified pPD49.26 with extra cloning sites [11]. The *lad-2* promoter contains a 4.0-kb sequence upstream of the start codon. Expression of the *lad-2::gfp* transcriptional reporter fusion was reported in the SMD, SAA, SDQ, ALN, and PLN neurons (Oliver Hobert, personal communication); we made independent strains that confirmed this expression pattern (unpublished data). The *lad-2::gcy-35* plasmid was injected at 50 ng/μl with 30 ng/μl *unc-122::dsRed* as a coinjection marker.

### Behavioral assays.

Aerotaxis assays were performed by placing animals on nematode growth medium (NGM) agar in custom-made microfluidic devices fabricated from polydimethylsiloxane (PDMS) with a gas-phase linear gradient from 0%–21% oxygen, and monitoring animals' accumulation in nine bins across the gradient [11]. Gradients were generated by delivering gases under laminar flow to source and drain chambers immediately outside the behavioral arena, using a syringe pump; diffusion of the gases established the gradient in the small assay chamber. Gases were obtained from Airgas (Radnor, Pennsylvania, United States), Matheson (Montgomeryville, Pennsylvania, United States), and TW Smith (Brooklyn, New York, United States). Animals were prepared for assays as described except that animals were picked instead of washed from their culture plate onto NGM assay plates before the devices were placed onto the assay plate. For assays on food, thin bacterial lawns of E. coli HB101 were made by seeding NGM plates for overnight growth at 37 °C and returning plates to room temperature for at least 1 h before assay.

Results from two devices were binned for one assay (~80–100 animals per assay). At least three independent assays on three different days for each genotype were used to generate results in the figures. Data presented represent distributions 25 min from start of the assay.

Glycerol osmotic avoidance assays for rescued *osm-9* strains were performed using a ring of 20 μl 4 M glycerol and xylene cyanol pipetted onto a NGM plate. The fraction of animals that escaped the ring was scored at 8 min after exposure. *osm-9; ADF::osm-9* animals were not rescued for osmotic avoidance (fraction escaping osmotic ring ± standard error of the mean for N2, 0.04 ± 0.02; for *osm-9,* 0.82 ± 0.06; and for *osm-9; ADF::osm-9,* 0.73 ± 0.06). *osm-9; osm-10(ASH, PHA, PHB)::osm-9* animals were partially rescued for osmotic avoidance (fraction escaping osmotic ring, 0.43 ± 0.06), suggesting variable expression of the transgene. To select for *osm-9; osm-10::osm-9* transgenic animals that strongly avoided high osmolarity, animals were washed and tested in the 4 M glycerol ring. Transgenic animals that stayed inside the ring after 8–10 min were picked off and recovered on a well-fed culture plate for at least 4 h before testing for aerotaxis.

### Statistical analysis.

Statistical analysis of aerotaxis was conducted in multiple steps. The nine individual points in the aerotaxis assay are not independent—accumulation of animals in one bin necessarily affects all other bins—so they cannot be analyzed individually. A first test to assess the overall distribution of animals in the aerotaxis assay was a comparison of experimental strains or conditions to controls by chi-square analysis (nine bins per aerotaxis assay × two strains = eight degrees of freedom). Chi-square analysis takes into account the non-Gaussian nature of the aerotaxis distributions as well as the total number of animals tested. It is overly conservative because it combines all animals tested into a single contingency table, and does not take into account the fact that each assay was repeated multiple times. Therefore, assays that are not statistically different by this criterion may actually be different. With this issue in mind, we have emphasized positive findings in this study. For controls such as N2 that were compared to multiple mutants, *p* < 0.01 was used as the level of significance for the chi-square test. Most results described in the paper were significant at *p* < 0.001 (Table S2). In the text, any stated differences in a secondary measure, such as hyperoxia avoidance index or median preferred oxygen concentration (see below), had also passed this first test using chi-square analysis of the entire distribution (*p* < 0.01).

The hyperoxia avoidance index has been previously described [11] and is defined as [(fraction of animals in 7%–14% O_2_) − (fraction of animals in 14%–21% O_2_)] / (fraction of animals in 7%–21% O_2_).

This standard performance index varies from −1.0 (all animals in hyperoxic regions) to +1.0 (all animals in normoxic regions), with 0 representing no preference between normoxia and hyperoxia. The response to hypoxia, the region between 0%–7% oxygen, was not reliably affected in any of the assays described here; hypoxia avoidance appears to be a distinct behavior with a distinct genetic basis, and this part of the curve was not informative for any analysis. Each strain or condition was tested in at least three assays with ~80–100 animals each, and the mean hyperoxia avoidance index across all assays was used to test significance. *t* tests were used to compare the hyperoxia avoidance indices between two distributions (Statview, Cary, North Carolina, United States). ANOVA plus Bonferroni *t* tests or Dunnett tests were used for multiple comparisons of hyperoxia avoidance indices between strains (Statview). For controls such as N2 that were compared to multiple mutants, *p* < 0.01 was used as the level of significance.

The median oxygen preference was calculated for each assay as the position of the 50th percentile of animals. Results from multiple assays were combined for each strain or condition using at least three assays with ~80–100 animals per assay, generating a mean median oxygen preference. For ANOVA, all strains were compared to one control (N2 in the absence of food), and Dunnett tests for multiple comparisons were used. Significant differences were observed with *gcy-34, gcy-36,* and *ocr-2* (all off food, higher oxygen preference than N2)*,* and *qaIs2241* off food, *npr-1* on food, and *daf-7* on food (lower oxygen preference than N2).

In almost every case, the hyperoxia avoidance index or the median oxygen preference uncovered and simplified the differences between strains and conditions that were apparent in the overall assays.

Two concerns remained after this analysis: (1) is it valid to consider the hyperoxia avoidance index and the median preferred oxygen concentration as independent values? and (2) are there important features of the behavior that were overlooked using these parameters? As an independent, unbiased way to identify important sources of variance in the data, we analyzed the distribution of animals in 279 aerotaxis assays by principal component analysis (PCA), an unsupervised data reduction method.

**Figure 9:**
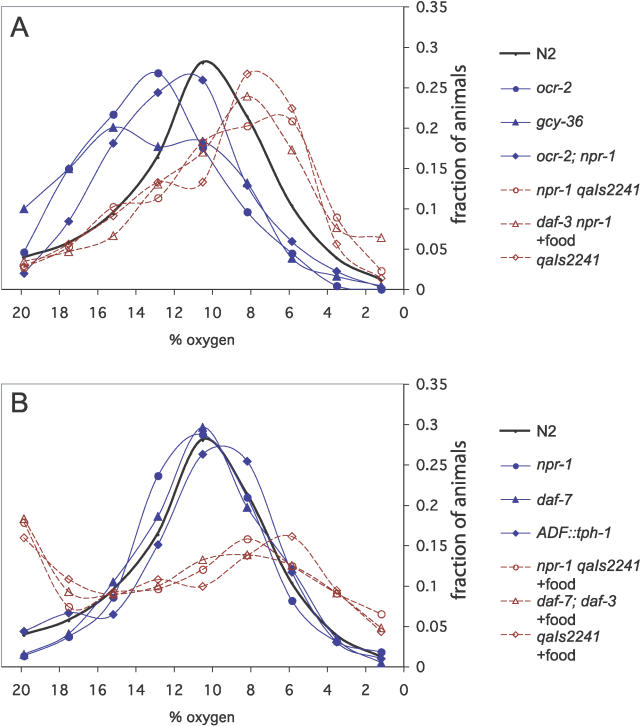
Second and Third Principal Components of Aerotaxis Data (PC2, PC3) (A) Assays with the highest (blue) and lowest (red) values for the second principal component (PC2), out of 36 sets of assays examined. Wild-type N2 animals off food are included for comparison (black, thick lines). (B) Assays with the highest (blue) and lowest (red) values for the third principal component (PC3), out of 36 sets of assays examined. Wild-type N2 animals off food are included for comparison (black, thick lines).

PCA was conducted on 48 datapoints that described each aerotaxis curve: the fraction of animals in each of the nine bins, the sums of fractions in adjacent bins or adjacent groups of three or four bins, and the differences between adjacent bins or adjacent groups of two or three bins. These 48 datapoints reflected features of the aerotaxis assay that were not necessarily represented in the standard parameters, such as the steepness of the curve at each point in the assay. Aerotaxis assays representing 36 genotypes with or without food were analyzed for PCA using Cluster 3.0 for Mac OSX (Michiel de Hoon, Seiya Imoto, and Satoru Miyano, University of Tokyo). The first principal component (PC1) described the basic bell shape of the aerotaxis assay, the second component (PC2) resembled the peak preferred oxygen concentration, and the third component (PC3) resembled the hyperoxia avoidance index. Each principal component represents a complex vector that is most easily understood by showing the assays with the highest and lowest value of that vector (Figure 9). For PC2, the highest values were the assays where the peak response was at high oxygen concentrations (e.g., *gcy-34, gcy-36, ocr-2*), and the lowest values were the assays in which the peak response was at low oxygen concentrations (e.g., *qaIs2241*); wild-type N2 animals had an intermediate value (Figure 9A). For PC3, the highest values were the assays with a strong aerotaxis peak (e.g., *npr-1, daf-7, ADF::tph-1*), and the lowest values were the assays that were nearly flat (e.g., N2-like genotypes on food); wild-type N2 animals off food had a high value (Figure 9B).

The conclusion of this analysis is that an unbiased assessment of the variance in the aerotaxis assays yields features that are substantially related to the parameters identified by direct inspection of the aerotaxis data: the hyperoxia avoidance index and the median preferred oxygen concentration. Since PC2 and PC3 are by definition independent of each other, it is valid to consider these two components of the aerotaxis assay (its peak and its strength) separately.

We reasoned that if the principal components defined in this manner were biologically significant, they would correlate with the genotype and condition of the assay. Therefore, after PCA of individual assays, assays were grouped by genotype and condition, and the mean and standard error for each principal component were defined for each strain and condition and subjected to ANOVA. This analysis demonstrated significant effects of genotype and condition on PC2 and PC3. Detailed statistical analysis of PC2 and PC3 by genotype and condition led to conclusions similar to those reported in the results (unpublished data).

## Supporting Information

Table S1 Distributions of Different Genotypes and Conditions in Aerotaxis Assays in a 0%–21% Oxygen Gradient Bins are equally spaced along the gradient as depicted in Figure 1A. Values are the fraction of animals in each bin. n, number of assays. Each assay represents 80–100 animals.
(218 KB PDF)

Table S2 Chi-Square Comparisons from This Study n, total number of animals tested for a given genotype and condition.
(410 KB PDF)

Table S3 Hyperoxia Avoidance on and off Food, and Aggregation on Food, of Different Genotypes (25 KB DOC)

## Abbreviations

ANOVA: analysis of variance
cGMP: cyclic guanosine monophosphate
NGM: nematode growth medium
PCA: principal component analysis
sGC: soluble guanylate cyclase homolog
TRPV: transient receptor potential channel, vanilloid
