# Supplementary material for: A Distributed Chemosensory Circuit for Oxygen Preference in C. elegans
Source: PLoS Biol. 2006 Aug 15;4(9):e274. doi: 10.1371/journal.pbio.0040274 (PMC1540710; doi:10.1371/journal.pbio.0040274)
Supplement: Supplementary file 1 [file pbio.0040274.st001.pdf]

Table S1. Distributions of different genotypes and conditions in aerotaxis assays in a 0-21% oxygen gradient.

Bins are equally spaced along the gradient as depicted in Figure 1A. Values are the fractions of animals in each bin. n, number of assays. Each assay represents 80-100 animals.

Genotype	Food	n	1	2	3	4	5	6	7	8	9
N2	off	28	0.039	0.057	0.094	0.163	0.280	0.214	0.104	0.039	0.011
gcy-32	off	3	0.043	0.060	0.139	0.164	0.203	0.231	0.119	0.032	0.010
gcy-34	off	4	0.034	0.078	0.201	0.230	0.232	0.134	0.068	0.021	0.002
gcy-35	off	9	0.062	0.117	0.156	0.140	0.176	0.147	0.112	0.070	0.019
gcy-36	off	6	0.103	0.150	0.199	0.172	0.189	0.124	0.045	0.014	0.004
osm-9	off	7	0.122	0.113	0.161	0.142	0.160	0.130	0.102	0.056	0.015
ocr-2	off	3	0.046	0.149	0.217	0.268	0.175	0.096	0.045	0.004	0.000
qaIs2241	off	8	0.028	0.056	0.091	0.132	0.133	0.267	0.224	0.056	0.013
gcy-35; qaIs2241	off	4	0.088	0.104	0.139	0.104	0.151	0.180	0.161	0.057	0.016
osm-9; qaIs2241	off	4	0.086	0.113	0.126	0.165	0.141	0.177	0.119	0.047	0.026
ocr-2; qaIs2241	off	4	0.119	0.094	0.109	0.110	0.134	0.150	0.176	0.072	0.037
tph-1; qaIs2241	off	3	0.085	0.129	0.120	0.151	0.152	0.158	0.159	0.024	0.021
npr-1	off	4	0.013	0.037	0.086	0.236	0.287	0.210	0.082	0.031	0.018
npr-1 qaIs2241	off	3	0.027	0.053	0.102	0.113	0.182	0.202	0.208	0.089	0.023
gcy-35; npr-1	off	3	0.068	0.097	0.122	0.208	0.173	0.168	0.089	0.055	0.020
osm-9; npr-1	off	3	0.087	0.114	0.184	0.156	0.195	0.148	0.083	0.022	0.012
ocr-2; npr-1	off	3	0.020	0.084	0.181	0.244	0.259	0.128	0.059	0.022	0.002
tph-1; npr-1	off	4	0.029	0.054	0.118	0.196	0.259	0.169	0.132	0.033	0.010
daf-3 npr-1	off	3	0.039	0.053	0.082	0.168	0.242	0.188	0.139	0.058	0.030
daf-7	off	4	0.015	0.041	0.105	0.186	0.296	0.197	0.121	0.035	0.005
daf-3	off	3	0.068	0.069	0.117	0.148	0.221	0.174	0.119	0.059	0.025
daf-7; daf-3	off	4	0.036	0.047	0.123	0.180	0.221	0.172	0.140	0.059	0.021
tax-4; kyIs342	off	4	0.054	0.111	0.105	0.153	0.188	0.188	0.120	0.049	0.031
ADF::tph-1	off	4	0.044	0.066	0.065	0.151	0.263	0.254	0.117	0.030	0.010
tph-1	off	11	0.075	0.109	0.123	0.129	0.204	0.197	0.117	0.037	0.009
tph-1; daf-3	off	3	0.065	0.088	0.125	0.167	0.213	0.175	0.099	0.048	0.020
gcy-35; SDQ::gcy-35	off	3	0.051	0.087	0.109	0.202	0.244	0.195	0.069	0.038	0.006
osm-9; ASH::osm-9	off	3	0.036	0.094	0.152	0.172	0.265	0.197	0.081	0.004	0.000
osm-9; ADF::osm-9	off	6	0.078	0.093	0.108	0.137	0.200	0.257	0.103	0.023	0.002
tph-1; ADF::tph-1	off	5	0.031	0.066	0.114	0.145	0.183	0.244	0.141	0.064	0.010
tph-1; NSM::tph-1	off	3	0.046	0.145	0.123	0.119	0.101	0.217	0.194	0.049	0.006
N2	on	10	0.152	0.109	0.119	0.152	0.154	0.120	0.088	0.071	0.035
gcy-35	on	3	0.254	0.109	0.089	0.120	0.172	0.116	0.070	0.044	0.026
gcy-36	on	3	0.204	0.096	0.128	0.128	0.183	0.087	0.087	0.063	0.025
osm-9	on	3	0.203	0.097	0.122	0.183	0.159	0.112	0.071	0.049	0.005
ocr-2	on	3	0.162	0.093	0.112	0.135	0.177	0.150	0.100	0.050	0.021
qaIs2241	on	4	0.159	0.108	0.089	0.108	0.099	0.137	0.161	0.095	0.043
npr-1	on	7	0.047	0.031	0.070	0.109	0.253	0.227	0.127	0.091	0.046
npr-1 qaIs2241	on	3	0.179	0.074	0.092	0.096	0.120	0.158	0.125	0.091	0.065
gcy-35; npr-1	on	4	0.181	0.099	0.116	0.131	0.151	0.127	0.101	0.077	0.017
osm-9; npr-1	on	3	0.142	0.089	0.140	0.134	0.189	0.122	0.092	0.064	0.028
ocr-2; npr-1	on	3	0.107	0.088	0.133	0.157	0.224	0.144	0.090	0.038	0.020
tph-1; npr-1	on	3	0.045	0.061	0.114	0.167	0.155	0.218	0.161	0.059	0.020
daf-3 npr-1	on	3	0.035	0.047	0.066	0.130	0.170	0.239	0.173	0.076	0.064
daf-7	on	6	0.038	0.058	0.086	0.139	0.188	0.212	0.147	0.084	0.048
daf-3	on	3	0.150	0.064	0.080	0.099	0.165	0.141	0.136	0.091	0.073
daf-7; daf-3	on	3	0.183	0.093	0.090	0.100	0.132	0.139	0.125	0.091	0.048
tax-4; kyIs342	on	3	0.052	0.085	0.109	0.117	0.155	0.212	0.195	0.048	0.025
tph-1	on	5	0.075	0.120	0.103	0.173	0.147	0.179	0.125	0.057	0.023
tph-1; daf-3	on	3	0.048	0.069	0.103	0.131	0.205	0.192	0.152	0.078	0.021
tph-1; ADF::tph-1	on	5	0.060	0.089	0.083	0.158	0.192	0.178	0.144	0.068	0.029
ADF::tph-1	on	5	0.078	0.073	0.095	0.123	0.175	0.198	0.127	0.092	0.040
