# Supplementary material for: A Distributed Chemosensory Circuit for Oxygen Preference in C. elegans
Source: PLoS Biol. 2006 Aug 15;4(9):e274. doi: 10.1371/journal.pbio.0040274 (PMC1540710; doi:10.1371/journal.pbio.0040274)
Supplement: Supplementary file 2 [file pbio.0040274.st002.pdf]

Table S2 (p. 1). Chi-square comparions from this study.
n, total number of animals tested for a given genotype and condition.

Group 1			Group 2			Chi-Square
Genotype	Food	n	Genotype	Food	n	
N2	off	2774	*gcy-36*	off	553	191 **
N2	off	2774	*gcy-35*	off	823	117 **
N2	off	2774	*gcy-34*	off	384	53 **
N2	off	2774	*gcy-32*	off	306	13
N2	off	2774	*qaIs2241*	off	801	121 **
N2	off	2774	*osm-9*	off	601	158 **
N2	off	2774	*ocr-2*	off	203	82 **
N2	off	2774	*tph-1*	off	1076	79 **
N2	off	2774	*npr-1*	off	414	20
N2	off	2774	*daf-7*	off	403	13
N2	off	2774	*daf-3*	off	289	18
N2	off	2774	*tax-4; kyIs342*	off	396	42 **
N2	off	2774	*ADF::tph-1*	off	422	8
gcy-35; qaIs2241	off	427	*qaIs2241*	off	801	44 **
gcy-35; qaIs2241	off	427	*gcy-35*	off	823	18
osm-9; qaIs2241	off	429	*qaIs2241*	off	801	76 **
osm-9; qaIs2241	off	429	*osm-9*	off	601	7
ocr-2; qaIs2241	off	378	*qaIs2241*	off	801	64 **
ocr-2; qaIs2241	off	378	*ocr-2*	off	203	76 **
tph-1; qaIs2241	off	282	*qaIs2241*	off	801	50 **
tph-1; qaIs2241	off	282	*tph-1*	off	1076	13
npr-1 qaIs2241	off	318	*npr-1*	off	414	51 **
npr-1 qaIs2241	off	318	*qaIs2241*	off	801	10
gcy-35; npr-1	off	311	*npr-1*	off	414	42 **
gcy-35; npr-1	off	311	*gcy-35*	off	823	12
osm-9; npr-1	off	364	*npr-1*	off	414	69 **
osm-9; npr-1	off	364	*osm-9*	off	601	16
ocr-2; npr-1	off	404	*npr-1*	off	414	39 **
ocr-2; npr-1	off	404	*ocr-2*	off	203	13
tph-1; npr-1	off	406	*npr-1*	off	414	13
tph-1; npr-1	off	406	*tph-1*	off	1076	34 **
daf-3 npr-1	off	274	*npr-1*	off	414	5
daf-3 npr-1	off	274	*daf-3*	off	289	19
daf-7; daf-3	off	388	*daf-7*	off	403	16
daf-7; daf-3	off	388	*daf-3*	off	289	6
tph-1; daf-3	off	303	*tph-1*	off	1076	8
tph-1; daf-3	off	303	*daf-3*	off	289	2
gcy-35; SDQ::gcy-35	off	325	*gcy-35*	off	823	31 **
gcy-35; SDQ::gcy-35	off	325	N2	off	2774	16
osm-9; ASH::osm-9	off	269	*osm-9*	off	601	52 **
osm-9; ASH::osm-9	off	269	N2	off	2774	28 **
osm-9; ADF::osm-9	off	532	*osm-9*	off	601	50 **
osm-9; ADF::osm-9	off	532	N2	off	2774	50 **
tph-1; ADF::tph-1	off	500	*tph-1*	off	1076	30 **
tph-1; ADF::tph-1	off	500	N2	off	2774	32 **
tph-1; NSM::tph-1	off	308	*tph-1*	off	1076	32 **
tph-1; NSM::tph-1	off	308	N2	off	2774	92 **

* $P < 0.01$
** $P < 0.001$

Table S2 (p. 2). Chi-square comparions from this study.

n, total number of animals tested for a given genotype and condition.

Group 1			Group 2			Chi-Square
Genotype	Food	n	Genotype	Food	n	
N2	off	2774	N2	on	848	248 **
gcy-35	off	823	*gcy-35*	on	240	81 **
qaIs2241	off	801	*qaIs2241*	on	346	103 **
osm-9	off	601	*osm-9*	on	249	19
ocr-2	off	203	*ocr-2*	on	254	49 **
npr-1	off	414	*npr-1*	on	627	44 **
npr-1 qaIs2241	off	318	*npr-1 qaIs2241*	on	243	49 **
gcy-35; npr-1	off	311	*gcy-35; npr-1*	on	294	26 **
osm-9; npr-1	off	364	*osm-9; npr-1*	on	337	19
ocr-2; npr-1	off	404	*ocr-2; npr-1*	on	308	41 **
tph-1; npr-1	off	406	*tph-1; npr-1*	on	312	19
daf-3 npr-1	off	274	*daf-3 npr-1*	on	281	13
daf-7	off	403	*daf-7*	on	589	47 **
daf-3	off	289	*daf-3*	on	197	22 *
daf-7; daf-3	off	388	*daf-7; daf-3*	on	233	58 **
tax-4; kyIs342	off	396	*tax-4; kyIs342*	on	311	12
tph-1	off	1076	*tph-1*	on	436	23 *
tph-1; daf-3	off	303	*tph-1; daf-3*	on	287	9
tph-1; ADF::tph-1	off	500	*tph-1; ADF::tph-1*	on	488	18
ADF::tph-1	off	422	*ADF::tph-1*	on	458	37 **
N2	on	848	*qaIs2241*	on	346	19
N2	on	848	*gcy-35*	on	240	19
N2	on	848	*osm-9*	on	249	15
N2	on	848	*ocr-2*	on	254	6
N2	on	848	*npr-1*	on	627	114 **
N2	on	848	*daf-7*	on	589	82 **
N2	on	848	*daf-3*	on	197	18
N2	on	848	*tax-4; kyIs342*	on	311	56 **
N2	on	848	*tph-1*	on	436	27 **
N2	on	848	*tph-1; daf-3*	on	287	40 **
N2	on	848	*tph-1; ADF::tph-1*	on	488	43 **
N2	on	848	*ADF::tph-1*	on	458	35 **
npr-1 qaIs2241	on	243	*npr-1*	on	627	61 **
npr-1 qaIs2241	on	243	*qaIs2241*	on	346	6
gcy-35; npr-1	on	294	*npr-1*	on	627	86 **
gcy-35; npr-1	on	294	*gcy-35*	on	240	8
osm-9; npr-1	on	337	*npr-1*	on	627	63 **
osm-9; npr-1	on	337	*osm-9*	on	249	15
ocr-2; npr-1	on	308	*npr-1*	on	627	53 **
ocr-2; npr-1	on	308	*ocr-2*	on	254	8
tph-1; npr-1	on	312	*npr-1*	on	627	34 **
tph-1; npr-1	on	312	*tph-1*	on	436	13
daf-3 npr-1	on	281	*npr-1*	on	627	18
daf-3 npr-1	on	281	*daf-3*	on	197	28 **
daf-7; daf-3	on	233	*daf-7*	on	589	54 **
daf-7; daf-3	on	233	*daf-3*	on	197	3
tph-1; daf-3	on	287	*tph-1*	on	436	14
tph-1; daf-3	on	287	*daf-3*	on	197	25 *

* $P < 0.01$
** $P < 0.001$
