# Supplementary material for: A Distributed Chemosensory Circuit for Oxygen Preference in C. elegans
Source: PLoS Biol. 2006 Aug 15;4(9):e274. doi: 10.1371/journal.pbio.0040274 (PMC1540710; doi:10.1371/journal.pbio.0040274)
Supplement: Supplementary file 3 [file pbio.0040274.st003.doc]

Table S3. Hyperoxia avoidance and aggregation behavior

| Genotype | | Hyperoxia Avoidance  (off food)^1^ | Hyperoxia Avoidance  (on food)^1^ | Aggregation (on food) | | | |  |
| --- | --- | --- | --- | --- | --- | --- | --- | --- |
| N2^2^ | | ++ | - | - | | | |  |
| *npr-1*^2^ | | ++ | ++ | ++ | | | |  |
| *gcy-35; npr-1*^3^ | | + | - | - | | | |  |
| *npr-1 qaIs2241* | | + | - | - | | | |  |
| *osm-9; npr-1*^4^ | | - | - | - | | | |  |
| *ocr-2; npr-1*^4^ | | + | - | - | | | |  |
| *daf-3 npr-1*^4^ | | ++ | ++ | ++ | | | |  |
| *tph-1; npr-1* | | ++ | + | + | | | |  |
| *daf-7* ^5^ | | ++ | ++ | ++ | | | |  |
| *daf-7; daf-3* ^5^ | | ++ | - | - | | | |  |
| *tph-1* | | + | + | - | | | |  |
| *tph-1; daf-3* | | + | + | - | | | |  |
| *tph-1; ADF::tph-1* | | ++ | + | - | | | |  |
| *ADF::tph-1* | | ++ | + | - | | | |  |
|  | |  |  |  | | | |  |
| ^1^ Hyperoxia avoidance index. ++, >0.45; +, 0.25-0.45; -, <0.25 | | | | | | | | |
| ^2^ Aggregation reported in [13]. | | | | | | |  | |
| ^3^ Aggregation reported in [16]. | | | | | | |  | |
| ^4^ Aggregation reported in [18]. | | | | | | |  | |
| ^5^ Aggregation reported in [19]. | | | | | | | | |
| All other results are from this study. | | | | | | | | |
|  | | | | | |  | | |
|  | | | | | |  | | |
|  |  | |  | |  | | | |
